# Major adverse cardiovascular events in people with chronic kidney disease in relation to disease severity and diabetes status

**DOI:** 10.1371/journal.pone.0221044

**Published:** 2019-08-28

**Authors:** Craig J. Currie, Ellen R. Berni, Thomas R. Berni, Sara Jenkins-Jones, Marvin Sinsakul, Lutz Jermutus, Philip Ambery, Meena Jain

**Affiliations:** 1 Global Epidemiology, Pharmatelligence, Cardiff, United Kingdom; 2 Institute of Population Medicine, School of Medicine, Cardiff University, Cardiff, United Kingdom; 3 Global Medical Affairs, MedImmune, AstraZeneca, Gaithersburg, Maryland, United States of America; 4 Cardiovascular Renal and Metabolism, MedImmune, AstraZeneca, Cambridge, United Kingdom; University of Colorado Denver School of Medicine, UNITED STATES

## Abstract

Diabetes plays an important role in the complex relationship between chronic kidney disease (CKD) and cardiovascular disease. This retrospective observational study compared the influence of estimated glomerular filtration rate (eGFR) and proteinuria on the risk of major adverse cardiovascular event (MACE; myocardial infarction or stroke) in CKD patients with and without diabetes. Data were from a linked database of UK electronic health records. Individuals with CKD and no prior MACE were classified as type 1 diabetes (T1DM; n = 164), type 2 diabetes (T2DM; n = 9,711), and non-diabetes (non-DM; n = 75,789). Monthly updated time-dependent Cox proportional hazard models were constructed to calculate adjusted hazard ratios (aHRs) for progression to MACE from first record of abnormal eGFR or proteinuria (index date). In non-DM, aHRs (95% CIs) by baseline eGFR category (referent G2) were G1: 0.70 (0.55–0.90), G3a: 1.28 (1.20–1.35), G3b: 1.64 (1.52–1.76), G4: 2.19 (1.98–2.43), and G5: 3.12 (2.44–3.99), and by proteinuria category (referent A1) were A2: 1.13 (1.00–1.28), A2/3 (severity indeterminable): 1.58 (1.28–1.95), and A3: 1.64 (1.38–1.95). In T2DM, aHRs were G1: 0.98 (0.72–1.32), G3a: 1.18 (1.03–1.34), G3b: 1.31 (1.12–1.54), G4: 1.87 (1.53–2.29), G5: 2.87 (1.82–4.52), A2: 1.22 (1.04–1.42), A2/3: 1.45 (1.17–1.79), and A3: 1.82 (1.53–2.16). Low numbers in T1DM precluded analysis. Modelling T2DM and non-DM together, aHRs were, respectively, G1: 3.23 (2.38–4.40) and 0.70 (0.55–0.89); G2: 3.18 (2.73–3.70) and 1.00 (referent); G3a: 3.65 (3.13–4.25) and 1.28 (1.21–1.36); G3b: 4.01 (3.40–4.74) and 1.65 (1.54–1.77); G4: 5.78 (4.70–7.10) and 2.21 (2.00–2.45); G5: 9.00 (5.71–14.18) and 3.14 (2.46–4.00). In conclusion, reduced eGFR and proteinuria were independently associated with increased risk of MACE regardless of diabetes status. However, the risk of MACE in the same eGFR state was 4.6–2.4 times higher in T2DM than in non-DM.

## Introduction

It has been estimated that 11–13% of the global population has some degree of chronic kidney disease (CKD),[1] as defined by international criteria based on structural damage and reduced function.[2,3]

A complex relationship exists between CKD and cardiovascular (CV) disease (CVD), with each being an established risk factor for the other.[4] CKD is an important risk factor for a wide variety of CVDs, including coronary heart disease and stroke,[5,6] even after accounting for the increased prevalence of traditional cardiometabolic risk factors in people with CKD. There is evidence that CV risk is increased at even the mildest levels of microalbuminuria,[7] and it is apparent that individuals with CKD are more likely to die of CVD than to progress to end stage renal disease.[8]

Glucose metabolism is integral to the pathophysiology of both diseases. Diabetes is the leading cause of CKD in all developed and most developing countries,[9] with approximately 20% of people with type 2 diabetes showing evidence of diabetic nephropathy within 20 years of diabetes onset,[10] and diabetes itself being an independent risk factor for CVD.[11] Whilst there is a J- or U-shaped association between hyperglycaemia—as measured by glycosylated haemoglobin (HbA1c)—and cardiovascular events,[12] the association between HbA1c and CKD risk is essentially a direct, linear association.[13]

With the current prevalence of diabetes estimated to be 9.4% of the adult population in the USA and 4.5% of the total population in the UK and the worldwide prevalence estimated to increase to 7.7% by 2030,[14–16] the public health impact of this triad of diseases is severe and increasing, accelerated by ageing populations and the rising prevalence of obesity and inactivity.

The purpose of this study, using a large linked data set of UK electronic health records, was to characterize the risk of and hospitalization rates for major adverse cardiovascular events (MACE), here defined as myocardial infarction or stroke, in a CKD population. In particular, this study compared the independent influences on this risk of two key aspects of kidney morbidity: decreased function as measured by estimated glomerular filtration rate (eGFR) and structural damage as evidenced by proteinuria, in people with and without diabetes.

## Methods

### Study design and data sources

This was a retrospective cohort study using primary care data from the UK Clinical Practice Research Datalink (CPRD) and linked inpatient data from the Hospital Episode Statistics (HES) for England.[17,18]

CPRD is an ongoing database of pseudonymized data collected in a non-interventional way from participating primary care practices throughout the UK. At July 2015, it contained research-quality electronic health records for more than 13 million patients registered at 689 practices. Data include demographics, diagnoses, symptoms, investigation and test results, referrals, and prescriptions. The records of more than 7 million CPRD patients (54%) registered with participating English practices can be linked, via a trusted third party, with other data sources, notably secondary care data from HES.

Clinical diagnoses, symptoms, and procedures in CPRD primary care data are recorded by Read Code (Version 2), a standard clinical terminology system used in UK general practice.[19] In HES data, diagnoses are encoded according to the 10th revision of the International Classification of Disease (ICD-10),[20] and procedures according to the UK Office of Population Censuses and Surveys Classification of Interventions and Procedures, version 4 (OPCS-4).[21] A number of studies have validated Read code recording in CPRD and its predecessor, the General Practice Research Database, showing a high positive predictive value overall.[22] CPRD patients are representative of the UK population in terms of age, gender, and ethnicity, and patients eligible for linkage to HES are in turn representative of the CPRD population.[17,23]

### Ethics

All patient data for the study were de-identified at source and then fully anonymized by a trusted third party on behalf of the CPRD before being made available to the researchers. Individual patients can opt out of contributing data to the CPRD. Approval for this study was granted by the CPRD Independent Scientific Advisory Committee (reference number 17_191R).

### Study population

The study population was drawn from patient records and practices classed by CPRD as being of an acceptable research quality and eligible for linkage to the HES data set. Individuals were selected if they had at least one diagnosis of chronic kidney disease (CKD) recorded in CPRD or HES and at least two abnormal eGFR measurements (<90 ml/min/1.73m^2^, indicative of decreased function; S1 Table) recorded between 91 and 730 days apart, adopting criteria used in the study by Schneider and colleagues.[24] The study index date was set as the earlier of the patient’s first abnormal eGFR record or, if available, first abnormal (increased) microalbuminuria or proteinuria record (as described in S2 Table). Individuals were required to have been registered at the practice for at least 365 days at index date and to have no prior record of dialysis, kidney transplant, renal cancer, myocardial infarction, or stroke.

Glomerular filtration rate values were estimated from serum creatinine test results by means of the CKD Epidemiology Collaboration (CKD-EPI) equation,[25] incorporating its adjustment for black ethnicity where this could be identified from the data source.

Tests for microalbuminuria and proteinuria (collectively termed proteinuria hereafter) are recorded in the CPRD primary care data in five different structured data areas (SDAs), three of which: ‘albumin–creatinine ratio’, ‘urine microalbumin’, and ‘urine biochemistry’ record both quantitative and qualitative results, while another two: ‘urinalysis—protein’ and ‘urine dipstick for protein’ record qualitative results only. Test data are accompanied by Read codes, which may provide more information about the nature of the test or of its result, and by data qualifiers (such as ‘normal’, ‘negative’, ‘+++’).

To address the challenge of identifying specific measures of proteinuria with appropriate units of measurement from the three quantitative SDAs, we extended the approach of Liang and colleagues by classifying each test SDA, unit of measurement, and accompanying Read code as specific, non-specific, or conflicting with respect to each of these measures in turn: albumin–creatinine ratio, protein–creatinine ratio, albumin excretion rate, protein excretion rate, spot albumin and spot protein.[26] Records were selected if at least one dimension was specific for a measure, with no conflicting dimension. Exceptions to this rule were: 1) where the Read code dimension was conflicting but the unit was specific; and 2) for spot albumin, where the title of the SDA was conflicting (‘albumin–creatinine ratio’) but the unit of measurement was specific.

Qualitative results were identified from the qualitative test SDAs and also from quantitative records that did not meet any of the rules listed above or had no numeric result entered. In addition, clinical records from CPRD and HES were included as qualitative results if their respective Read or ICD-10 codes indicated the absence or presence of proteinuria.

In accordance with the Kidney Disease: Improving Global Outcomes (KDIGO) clinical practice guidelines,[3] the severity of chronic kidney disease was classified into six categories, from G1: normal or high to G5: kidney failure, based on the person’s eGFR measurements (S1 Table). Classification of proteinuria severity was also based on a KDIGO classification,[3] from A1: normal to mildly increased to A3: severely increased, with a fourth category, A2/3, added here to encompass qualitative results from which the severity of abnormality could not be determined (S2 Table).

The selected individuals were classified into three cohorts: type 1 diabetes (T1DM), type 2 diabetes (T2DM), and non-diabetes (non-DM). Individuals were entered in the T2DM cohort if they had one or more diagnostic record for type 2 diabetes or one or more prescription for a glucose-lowering drug other than insulin, and had no record of secondary diabetes nor, if identified by metformin prescription alone, of polycystic ovarian syndrome. Individuals were entered in the T1DM cohort if they had one or more prescription for insulin but none for any other glucose-lowering drug, had no diagnoses of type 2 diabetes, and at least one of the following criteria applied:

- At least one diagnostic record of type 1 diabetes using a diagnostic code other than a Read code for ‘insulin-dependent diabetes mellitus’ (a term that may be misapplied to a person with type 2 diabetes receiving insulin therapy)
- At least one diagnostic record of type 1 diabetes and aged 30 years or younger at diabetes presentation (earlier of first insulin or first diagnosis)
- At least one diagnostic record of type 1 diabetes, aged 31 to 39 at diabetes presentation, and a body mass index (BMI) at presentation ≤ 25 kgm^-2^.

Individuals were entered in the non-DM cohort if they had no record of diabetes or glucose-lowering therapy in the data source and had no more than one record of HbA1c ≥ 6.5% (47.5 mmol/mol).

Individuals in the two diabetes cohorts were required to have a diabetes presentation date on or before their index date. Individuals in the T2DM and non-DM cohorts were required to be aged 40 years or older at index date; those in the T1DM cohort were required to be 25 years or older at index.

End of data follow-up was calculated as the earliest of: the person’s death or transfer-out date, their practice’s last data-collection date, their individual HES linkage date, or the end of the linkage scheme (31 March 2014). Data censoring was also invoked at a person’s first record, post index date, of dialysis, renal transplant, or renal cancer.

### Study endpoints

The endpoints of this study were calculated by diabetes cohort and comprised:

- Unadjusted time to first MACE, stratified by eGFR and proteinuria category
- Adjusted risk of first MACE by eGFR and proteinuria category
- Event rates for hospital admissions for MACE

The composite outcome of first MACE was identified as the individual’s first record from primary care or HES inpatient data having a diagnostic code for myocardial infarction or stroke. Hospitalizations for MACE were identified from HES inpatient data as distinct admissions having a diagnosis of myocardial infarction or stroke.

The outcomes of dialysis and transplant were determined by Read code in CPRD or by ICD-10 or OPCS-4 procedural code in HES inpatient data. Baseline values for BMI, weight, height, systolic and diastolic blood pressure, serum creatinine, and—for the diabetes cohorts—HbA1c were identified from the nearest record to index date, provided this was no more than 365 days before or 30 days after index date and searching in the following order: -30 days, +30 days, -365 days. Baseline smoking and alcohol status were identified from the nearest record prior to index date; if no such status was recorded, the nearest status following index date was used.

### Statistical methods

Baseline characteristics for each of the three cohorts: non-DM, T1DM, and T2DM were summarized using mean and standard deviation or median and interquartile range, depending on their distribution.

For each cohort and for an ‘overall’ cohort combining all T1DM, T2DM, and non-DM individuals, Kaplan–Meier curves for unadjusted times to first MACE were produced, stratified by baseline eGFR and by proteinuria category at or nearest to MACE (proteinuria at baseline was too sparse to enable stratification by these values).

Cox proportional hazard models were then applied to the individual and combined cohorts to estimate the adjusted hazard ratios (aHR) for first MACE, with 95% confidence intervals (CI). In these time-dependent models, eGFR and proteinuria levels were updated on a monthly basis, applying last-observation-carried-forward (preferred) or next-observation-carried-backward techniques where necessary and where, in the case of proteinuria, a patient had ≥1 proteinuria measurement (all patients had ≥2 eGFR measurements). The parameterization of each covariate is outlined in Table 1.

**Table 1:** Baseline characteristics for the type 1 diabetes (T1DM), type 2 diabetes (T2DM) and non-diabetes (non-DM) cohorts.

Characteristic	Non-DM		T2DM		T1DM		Overall	
N (%)	75,789	(88.5)	9,711	(11.3)	164	(0.2)	85,664	(100)
Age, years, mean (sd)	69.2	(11.3)	66.6	(10.4)	46.5	(13.0)	68.8	(11.3)
Male, n (%)	28,985	(38.2)	4,950	(51.0)	95	(57.9)	34,030	(39.7)
Duration of diagnosed diabetes, years, median (IQR)	—	(—)	3.1	(0.7–8.4)	20.1	(14.0–30.4)	3.2	(0.8–8.7)
Duration of diagnosed CKD, years, median (IQR)	0	(0–0)	0	(0–0)	0	(0–0)	0	(0–0)
Duration of diagnosed renal disease, years, median (IQR)	4.3	(2.2–6.7)	4.8	(2.8–7.1)	3.3	(0.1–5.8)	4.4	(2.3–6.7)
BMI, kgm^-2^, mean (sd)	27.6	(5.1)	30.1	(6.0)	25.8	(3.9)	28.1	(5.4)
Weight, kg, mean (sd)	75.9	(16.2)	84.4	(18.6)	76.8	(13.3)	77.8	(17.1)
Height, m, mean (sd)	1.7	(0.1)	1.7	(0.1)	1.7	(0.1)	1.7	(0.1)
Systolic BP, mmHg, mean (sd)	149.3	(21.3)	146.9	(19.8)	139.2	(20.9)	149.0	(21.1)
Diastolic BP, mmHg, mean (sd)	83.8	(11.6)	81.5	(10.8)	78.9	(11.5)	83.5	(11.5)
Smoking, n (%)								
Never	43,702	(57.7)	5,231	(53.9)	90	(54.9)	49,023	(57.2)
Prior smoker	19,385	(25.6)	2,748	(28.3)	28	(17.1)	22,161	(25.9)
Current smoker	12,125	(16)	1,701	(17.5)	45	(27.4)	13,871	(16.2)
Missing	577	(0.8)	31	(0.3)	1	(0.6)	609	(0.7)
Alcohol, n (%)								
Never	14,449	(19.1)	2,412	(24.8)	23	(14.0)	16,884	(19.7)
Prior drinker	1,376	(1.8)	277	(2.9)	6	(3.7)	1,659	(1.9)
Current drinker	55,086	(72.7)	6,624	(68.2)	123	(75)	61,833	(72.2)
Missing	4,878	(6.4)	398	(4.1)	12	(7.3)	5,288	(6.2)
HbA1c, %, median (IQR)	5.7	(5.4–6.0)	7.6	(6.6–9.0)	9.0	(8.0–10.5)	7.4	(6.3–8.9)
HbA1c, mmol/mol, median (IQR)	38.8	(35–42)	59	(49–75)	75	(64–91)	57	(45–74)
Serum creatinine, μmol/l, mean (SD)	103.7	(31.5)	99.2	(28.2)	116.2	(60.7)	103.2	(31.3)
GP contacts in prior year, n, median (IQR)	5	(3–9)	8	(4–14)	7	(3–12)	5	(3–9)
History of peripheral vascular disease, n (%)	628	(0.8)	336	(3.5)	12	(7.3)	976	(1.1)
eGFR category, n (%)*								
G2	38,892	(51.3%)	6,003	(61.8%)	92	(56.1%)	44,987	(52.5%)
G3a	26,599	(35.1%)	2,193	(22.6%)	14	(8.5%)	28,806	(33.6%)
G3b	8,203	(10.8%)	731	(7.5%)	12	(7.3%)	8,946	(10.4%)
G4	1,657	(2.2%)	175	(1.8%)	10	(6.1%)	1,842	(2.2%)
G5	248	(0.3%)	16	(0.2%)	2	(1.2%)	266	(0.3%)
Missing	190	(0.3%)	593	(6.1%)	34	(20.7%)	817	(1.0%)
Proteinuria category, n (%)**								
A1	23,650	(31.2%)	4,325	(44.5%)	57	(34.8%)	28,032	(32.7%)
A2	7,319	(9.7%)	2,335	(24.0%)	29	(17.7%)	9,683	(11.3%)
A2/3	453	(0.6%)	303	(3.1%)	7	(4.2%)	762	(0.9%)
A4	1,721	(2.3%)	1,121	(11.5%)	40	(24.4%)	2,882	(3.4%)
Missing	42,647	(56.3%)	1,627	(16.8%)	31	(18.9%)	44,305	(51.7%)

IQR, interquartile range; BMI, body mass index; BP, blood pressure; HbA1c, glycosylated haemoglobin

* At baseline

** Based on nearest record prior to earlier of first major adverse cardiovascular event or data censoring

In the first set, models were applied to three cohorts: T2DM, non-DM, and the overall cohort (including T1DM), adjusting for the separate effects of eGFR category (compared with the referent category G2), proteinuria category (referent A1), and the potential confounding covariates of baseline age at the index date, gender, baseline BMI, baseline blood pressure, smoking status, index year, immunosuppression status, and prior comorbidities. Additionally, in the T2DM cohort, the model was adjusted for baseline HbA1c and, in the overall cohort, for baseline HbA1c and diabetes cohort membership. Separate analyses for the T1DM cohort were not carried out because of small numbers. G2 was chosen as the referent category for the eGFR analyses because of small numbers in the G1 category.

The second set of models was applied to the T2DM and non-DM cohorts together to consider how eGFR category affected the risk of MACE between these two larger cohorts. Adjusted hazard ratios for time to first MACE were presented by diabetes status and eGFR group, with the G2 group in non-DM individuals as the referent and adjusting for the potentially confounding covariates listed above. As a post-hoc analysis, we considered the results for non-DM and T2DM separately by gender.

The third set of models considered the effects of eGFR and proteinuria interaction on the risk of MACE in the combined T1DM, T2DM, and non-DM cohort. The G1 eGFR category in combination with the A1 proteinuria category was used as the referent, and models were additionally adjusted for gender, cohort, and baseline age, smoking status, and blood pressure.

Event rates for hospitalizations for MACE were calculated per 1,000 person years (PKPY) follow-up (to censor date).

## Results

### Baseline characteristics

There were 75,789 people in the non-DM cohort, 9,711 in the T2DM, and 164 in the T1DM. Table 1 details the baseline characteristics for each cohort. Individuals in the non-DM cohort had the highest mean age (69.2 years), followed by those in the T2DM cohort (66.6 years) and then the T1DM cohort (46.5 years). The non-DM cohort was 38.2% male, the T2DM cohort 51.0% male, and the T1DM cohort 57.9% male. The median duration of diagnosed diabetes at index date was higher in T1DM (20.1 years) than in T2DM (3.1 years), which was to be expected. Median duration of diagnosed renal disease was highest in T2DM (4.8 years) and lowest in T1DM (3.3 years). BMI was lowest in T1DM (25.8 kg/m^2^) and highest in T2DM (30.1 kg/m^2^). The proportion of prior (ex-) smokers was highest in T2DM (28.3%) and lowest in T1DM (17.1%), although the T1DM cohort had the highest proportion of current smokers (27.4%). Median HbA1c was higher in the T1DM cohort, at 9.0% (75 mmol/mol), than in the T2DM cohort, where it was 7.6% (59 mmol/mol). Understandably, there was marked variability between the cohorts.

### Unadjusted time to first major adverse cardiovascular event

**Fig 1:**
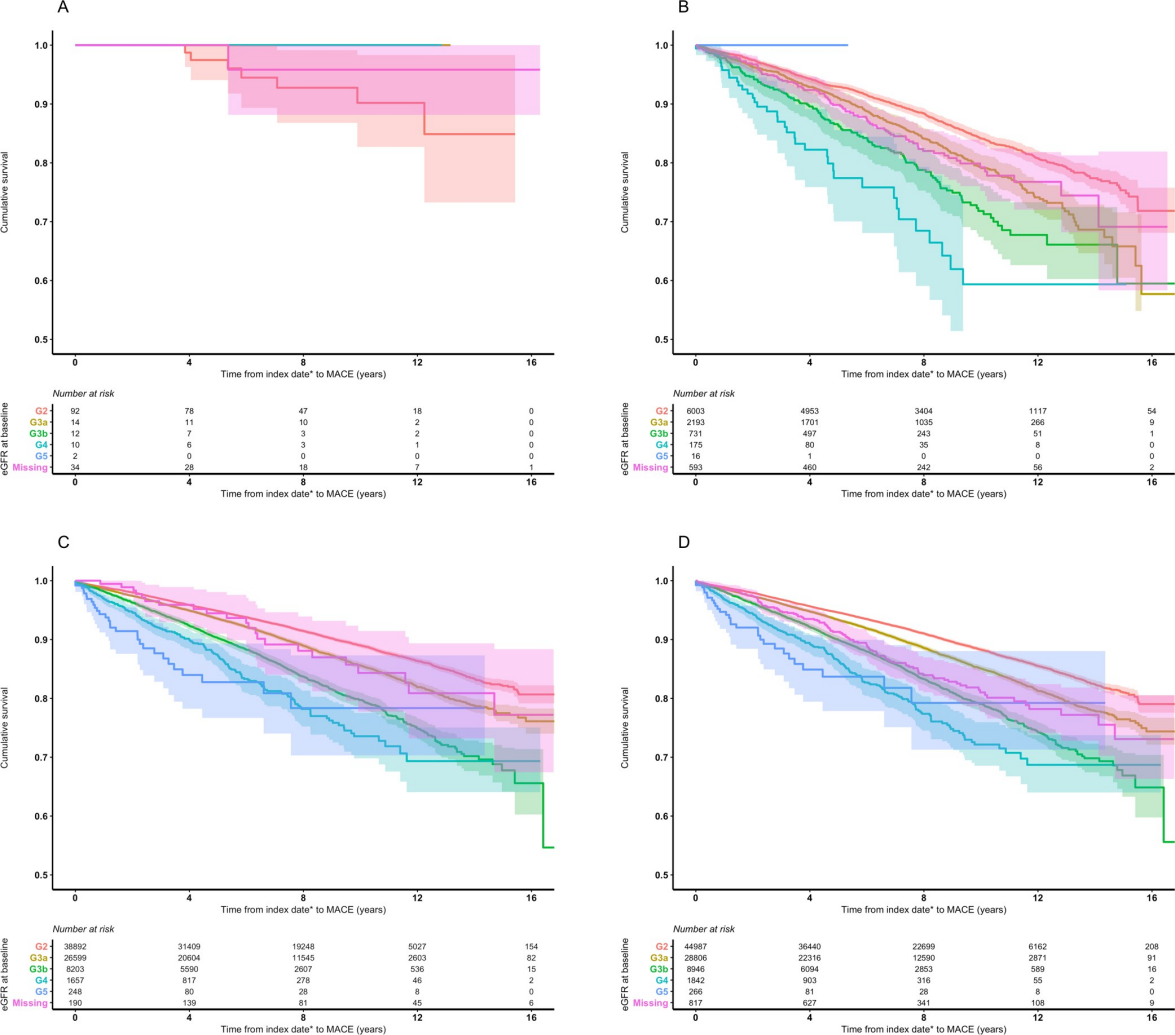
Kaplan–Meier plots, with 95% confidence intervals, for time to first major adverse cardiovascular event by diabetes cohort and baseline eGFR category. **a. Type 1 diabetes, b. Type 2 diabetes, c. Non-diabetes, d. Overall** MACE, major adverse cardiovascular event. eGFR, estimated glomerular filtration rate. * Index date defined as the earlier of the patient’s first abnormal eGFR or, if available, first abnormal proteinuria record.

**Fig 2:**
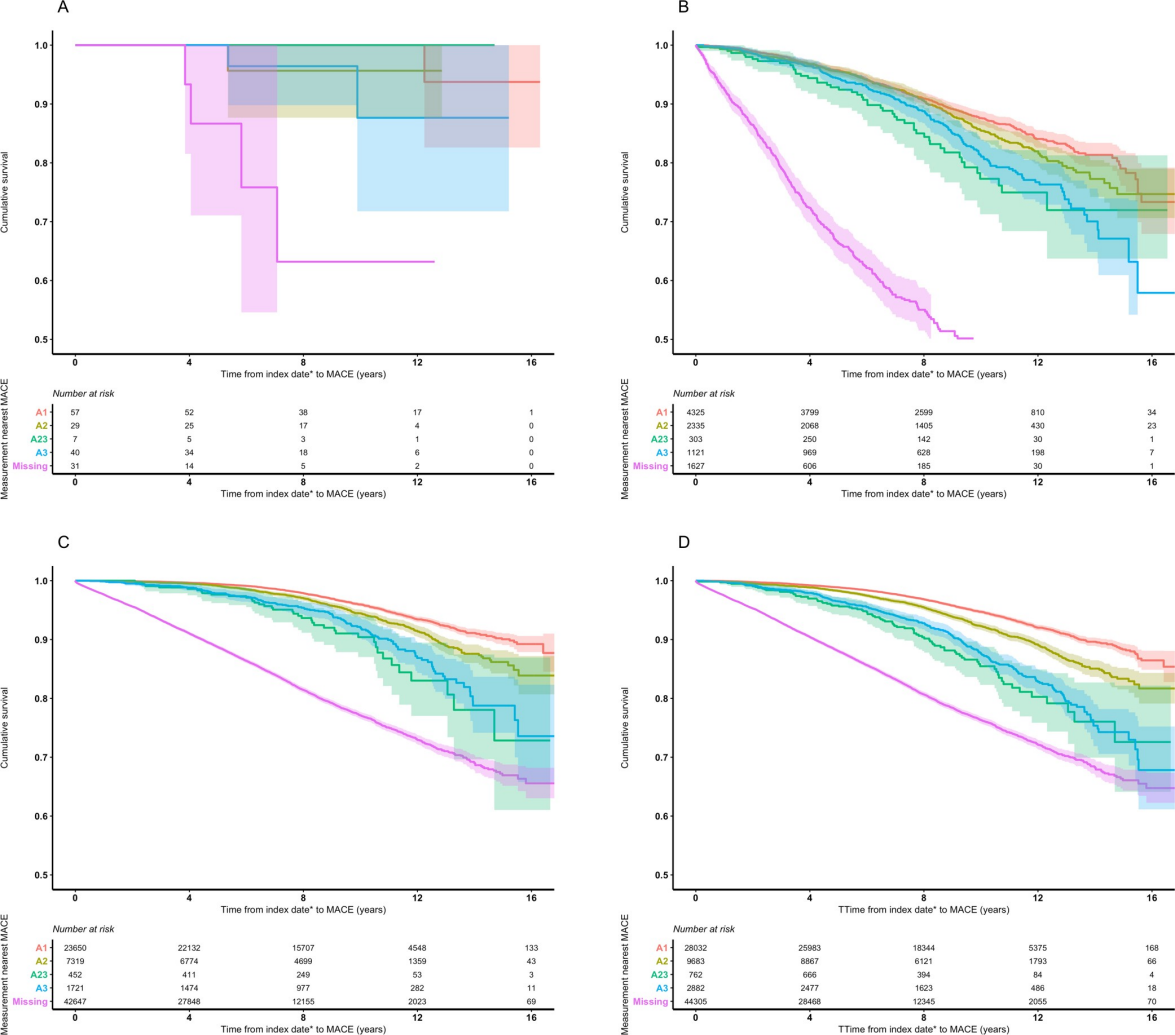
Kaplan–Meier plots, with 95% confidence intervals, for time to first major adverse cardiovascular event by diabetes cohort and nearest proteinuria category prior to event. **a. Type 1 diabetes, b. Type 2 diabetes, c. Non-diabetes, d. Overall** MACE, major adverse cardiovascular event. * Index date defined as the earlier of the patient’s first abnormal eGFR or, if available, first abnormal proteinuria record.

Kaplan–Meier curves showing the time to first MACE event for each cohort, stratified separately by eGFR and proteinuria categories, are illustrated in Figs 1 and 2, respectively.

The T1DM cohort comprised only a small number of people, and therefore its Kaplan–Meier curves are difficult to interpret.

When the T2DM cohort was stratified by eGFR category, <50% of people experienced an event within the follow-up period in all categories; therefore, the median could not be calculated. The upper quartile of survival was reached in G2 (15.1 years, 95%CI 14.2–NA), G3a (11.6, 11.1–13.1), G3b (9.0, 8.1–14.8), and G4 (6.9, 4.6–8.9) (Fig 1B). When this cohort was stratified by proteinuria category, the upper quartile of survival was reached in A1 (15.5, 15.4–NA), A2 (14.8, 13.8–NA), A2/3 (10.7, 11.3–14.1), and A3 (12.9, 11.3–14.1) (Fig 2B).

There was a discernible difference between eGFR categories in non-DM (Fig 1C). The upper quartiles of survival were 12.0 years (95%CI 11.3–12.6) in category G3b, 9.4 (8.2–NA) in G4, and the upper quartiles of survival were not reached for those with a measurement in G5. There was a clear distinction between proteinuria categories after eight years (Fig 2C). People in proteinuria categories A2/3 and A3 (who shared a similar pattern) progressed to MACE at the most rapid rate, followed by those in category A2 and then A1.

In the overall cohort, comprising all three cohorts together, and stratifying by eGFR category, the upper quartile of survival was 15.6 years for G3a (95%CI 15.2– NA), 11.7 (11.0–12.4) for G3b, 8.9 (7.9–10.9) for G4, and was not reached for G5 (Fig 1D). When the merged cohorts were stratified by proteinuria category, the upper quartile of survival was reached in A2/3 (14.7 years, 12.3–NA) and A3 (14.1, 13.6–NA) (Fig 2D).

Across all cohorts, patients in the ‘missing’ proteinuria category progressed more rapidly to MACE than did patients with known proteinuria status.

### Adjusted risk of progression to first major adverse cardiovascular event

**Fig 3:**
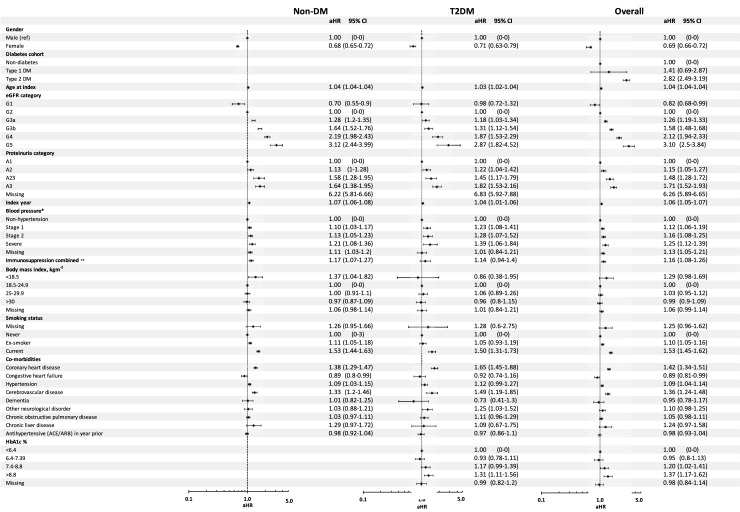
Adjusted hazard ratios for first major adverse cardiovascular event in type 2 diabetes, non-diabetes, and overall (combined types 1 and 2 diabetes and non-diabetes) cohorts. * Non-hypertension = systolic < 140, or diastolic < 90; stage 1 = 140 ≤ systolic blood pressure < 160, or 90 ≤ diastolic < 100; stage 2 = 160 ≤ systolic < 180, or 100 ≤ diastolic < 110; severe = systolic ≥ 180, or diastolic ≥ 110 (units = mmHg). ** Patient has a prior record of immunosuppressive disease or was prescribed an immunosuppressive therapy in the year prior to baseline. aHR, adjusted hazard ratio. CI, confidence interval. DM, diabetes. Non-DM, non-diabetes. T2DM, type 2 diabetes. ACE, angiotensin-converting enzyme inhibitor. ARB, angiotensin II receptor antagonist. HbA1c, glycosylated haemoglobin.

Applying Cox proportional hazards models to the merged cohorts (T1DM, T2DM, and non-DM) revealed a significant difference in adjusted MACE risk between eGFR category G1 and the referent G2 (adjusted hazard ratio (aHR) = 0.82, 95%CI 0.68–0.99) (Fig 3). All other eGFR categories showed an increased risk of MACE compared with G2: people in category G3a had an aHR of 1.26 (1.19–1.33); in G3b, this was 1.58 (1.48–1.68); in G4, 2.12 (1.94–2.33); and in G5, 3.10 (2.50–3.84). There were also differences in adjusted MACE rates between proteinuria category A2 (aHR = 1.15, 95%CI 1.05–1.27), A2/3 (1.48, 1.28–1.72), and A3 (1.71, 1.52–1.93) by comparison with the referent A1 (Fig 3). Prior coronary heart disease (1.42, 1.34–1.51), hypertension (1.09, 1.04–1.14), and cerebrovascular disease (1.36, 1.24–1.48) resulted in an increased aHR. Ex-smokers and current smokers had an increased risk of MACE of 10% (1.10, 1.05–1.16) and 53% (1.53, 1.45–1.62), respectively. BMI was not significant in the model. People with severe blood pressure has the highest risk of MACE when compare with non-hypertension (1.25, 1.12–1.39), followed by stage 2 (1.16, 1.08–1.25) and then stage 1 hypertension (1.12, 1.06–1.19). By comparison with the non-DM cohort, people in T2DM had an increased risk of MACE (2.82, 2.49–3.19). Males had an increased risk of MACE compared with females.

### Adjusted risk of progression to first major adverse cardiovascular event by diabetes status

Separate models were repeated for the T2DM and non-DM cohorts. (The proposed models were inappropriate for the T1DM cohort because of the low cohort membership.) Within T2DM, there was no significant difference between eGFR category G1 (aHR = 0.98, 95%CI 0.72–1.32) compared with the referent G2 (Fig 3). There was an association between worsening eGFR and MACE rates in categories G3a (1.18, 1.03–1.34), G3b (1.31, 1.12–1.54), G4 (1.87, 1.53–2.29), and G5 (2.87, 1.82–4.52) compared with G2.

A similar pattern of association was seen in the results from the non-DM cohort (Fig 3), where adjusted rates were higher in eGFR category G3a (aHR = 1.28, 95%CI 1.20–1.35), G3b (1.64, 1.52–1.76), G4 (2.19, 1.98–2.43), and G5 (3.12, 2.44–3.99) compared with the referent G2. The only difference was the G1 category, where there was a greater reduction in risk of MACE compared with G2 than in the T2DM cohort (0.70, 0.55–0.90) (Fig 3).

Within the T2DM cohort, differences in adjusted risk between the various proteinuria categories were significant: A2 (aHR = 1.22, 95%CI 1.04–1.42), A2/3 (1.45, 1.17–1.79), and A3 (1.82, 1.53–2.16) compared with the referent A1. A similar pattern was seen in the non-DM cohort: A2 (1.13, 1.00–1.28), A2/3 (1.58, 1.28–1.95), and A3 (1.64, 1.38–1.95) compared with A1 (Fig 3). Adjusted hazard ratios for the other covariates are detailed.

**Fig 4:**
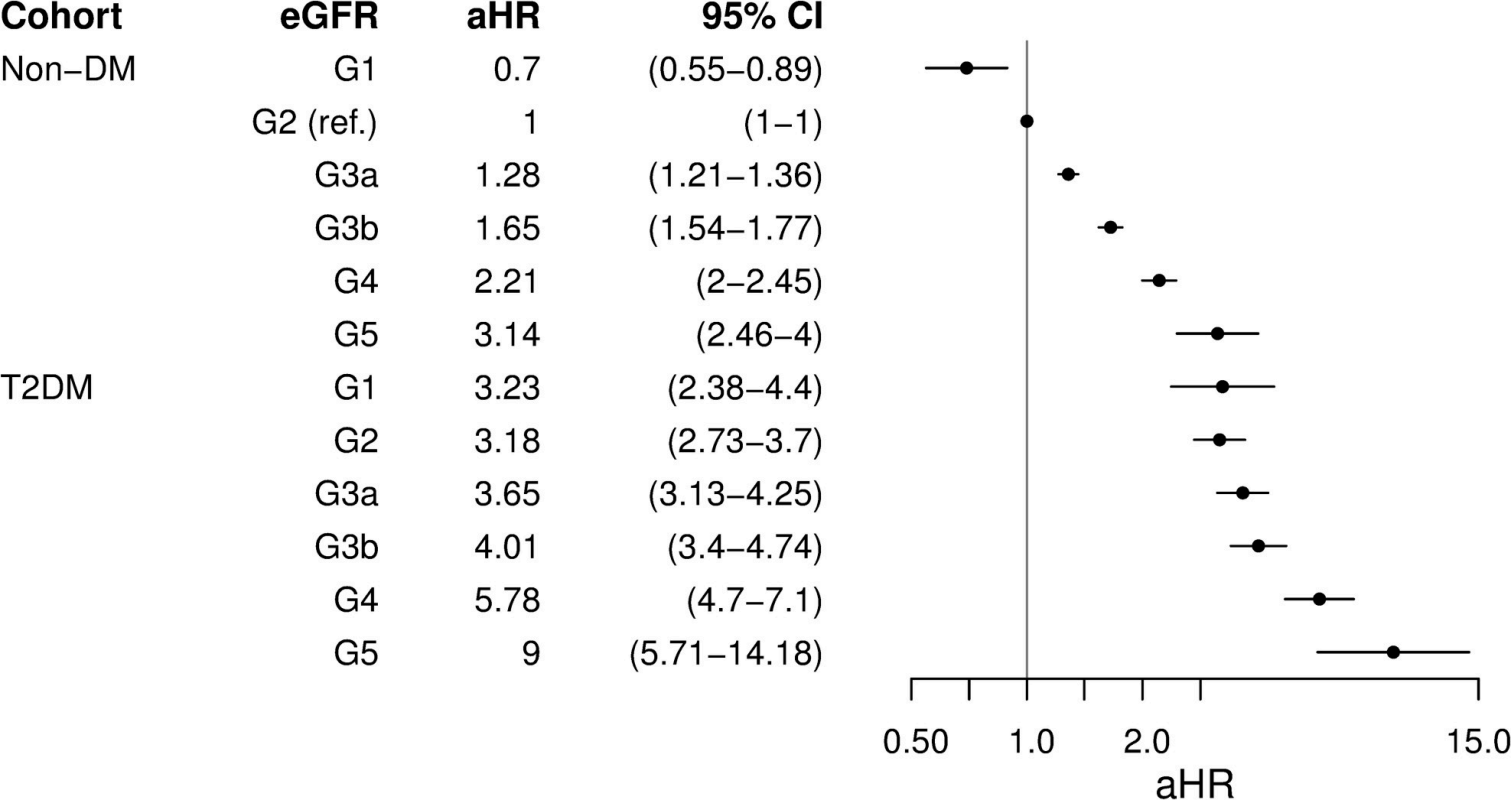
Adjusted hazard ratios for first major adverse cardiovascular event by eGFR category and diabetes status in Cox models combining type 2 and non-diabetes cohorts. eGFR, estimated glomerular filtration rate. aHR, adjusted hazard ratio. CI, confidence interval. Non-DM, non-diabetes. T2DM, type 2 diabetes. aHR, adjusted hazard ratio.

In a second set of models adjusting by eGFR category and diabetes status in the combined T2DM and non-DM cohorts, with the G2 category in non-DM as the referent, there was an increase in aHRs within each diabetes cohort as the eGFR category increased (Fig 4). All eGFR categories in the T2DM cohort had a significantly higher aHR than did the corresponding eGFR category in the non-DM cohort: G1: 3.23 (95% CI 2.38–4.40) versus 0.70 (0.55–0.89) in non-DM (ratio 4.6); G2: 3.18 (2.73–3.70) versus 1.00 (referent; ratio 3.2); G3a: 3.65 (3.13–4.25) versus 1.28 (1.21–1.36; ratio: 2.9); G3b: 4.01 (3.40–4.74) versus 1.65 (1.54–1.77; ratio: 2.4); G4: 5.78 (4.70–7.10) versus 2.21 (2.00–2.45; ratio: 2.6); and G5: 9.00 (5.71–14.18) versus 3.14 (2.46–4.00; ratio: 2.9), respectively.

**Fig 5:**
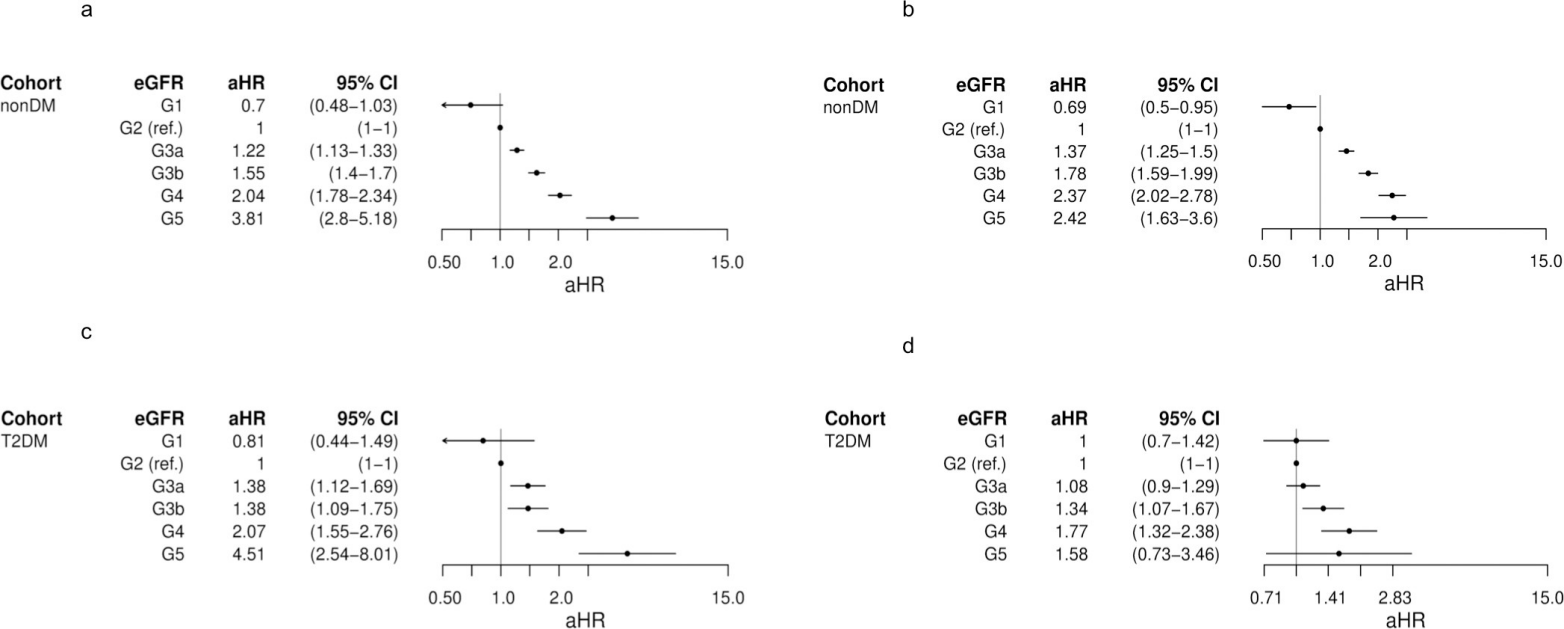
Adjusted hazard ratios for first major adverse cardiovascular event by time-dependent eGFR category in Cox models for type 2 and non-diabetes cohorts split by gender. **a. Non-diabetes–female, b. Non-diabetes–male, c. Type 2 diabetes–female, d. Type 2 diabetes–male** eGFR, estimated glomerular filtration rate. aHR, adjusted hazard ratio. CI, confidence interval. Non-DM, non-diabetes. T2DM, type 2 diabetes.

A *post-hoc* analysis considered the effect that gender had on MACE risk. There was a similar trend observed amongst both males and females in each cohort for eGFR severity categories G1 to G4, inclusive (Fig 5). However, there was a differing pattern in the G5 category, whereby in the T2DM cohort, females had an increased risk of MACE with an aHR of 4.51 (95% CI 2.54–8.01) vs. males who had an aHR of 1.58 (0.73–3.46). A similar pattern was seen in the non-DM cohort, where females had an aHR of 3.81 (2.80–5.18) vs. males, who had an aHR of 2.42 (1.63–3.6).

#### Adjusted risk of progression to first major adverse cardiovascular event by combined eGFR and proteinuria status

**Fig 6:**
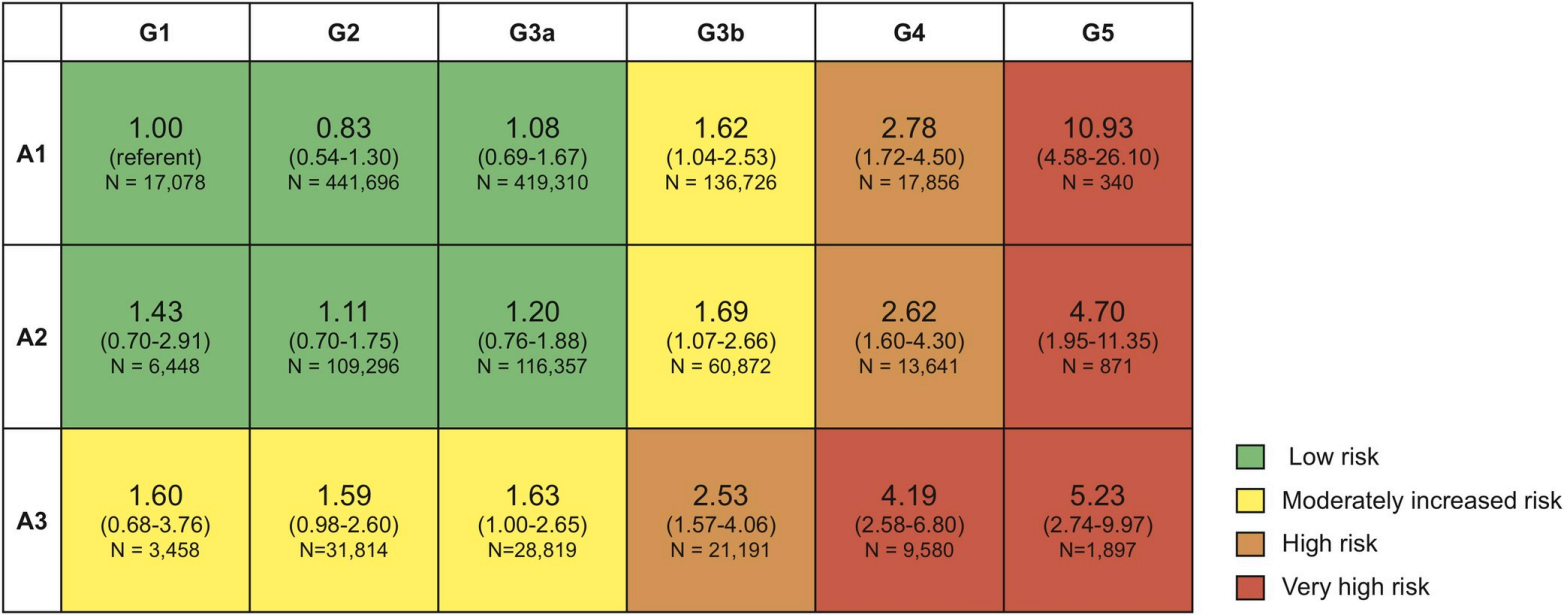
Adjusted hazard ratios for first major adverse cardiovascular event by combined eGFR and proteinuria categories in Cox models combining type 1, type 2, and non-diabetes cohorts. Low risk: adjusted hazard ratio (aHR) 1–1.5, moderately increased risk: aHR 1.5–2.5, high risk: aHR 2.51–3.5, very high risk: aHR >3.5; 95% confidence intervals are shown in parentheses. N, number of patient months.

There was a consistent pattern when models were applied adjusting for the interaction between eGFR and proteinuria in the overall cohort (Fig 6). All G5 combinations with any proteinuria status (including the normal level A1) showed very high risk of MACE (aHR>3.5), with people combining G5/A1, G5/A2, and G5/A3 having aHRs of 10.93 (95% CI 4.58–26.10), 4.70 (1.95–11.35), and 5.23 (2.74–9.97), respectively. Adjusted risk of MACE was also very high in the combination G4/A3 (4.19, 2.58–6.8) and high (2.5<aHR≤3.5) in G4/A1 (2.78, 1.72–4.5), G4/A2, (2.62, 1.60–4.30), and G3b/A3 2.53 (1.57–4.06). There was a moderately increased adjusted risk (1.5<aHR≤2.5) of progressing to MACE in all other combinations of proteinuria status with G3b: G3b/A1 (1.62, 1.04–2.53) and G3b/A2 (1.69, 1.07–2.66) and in all other eGFR combinations with severe proteinuria A3: A3/G1 (1.60, 0.68–3.76), A3/G2 (1.59, 0.98–2.60), and A3/G3a (1.63, 1.00–2.65).

### Hospitalization rates for major adverse cardiovascular events

Rates of hospitalization for MACE were higher in T2DM, at 18.4 admissions per 1,000 person years (PKPY), than in non-DM (13.5 PKPY) and T1DM (6.1 PKPY) (Table 2). When people in non-DM were stratified by eGFR category at baseline, the crude event rates were highest in G5 (kidney failure; 32.4 PKPY) followed by G4 (27.3 PKPY), G3b (21.0 PKPY), G3a (14.4 PKPY), and G2 (11.2 PKPY). A similar pattern was seen in T2DM, where the crude event rates were highest in G4 (43.8 PKPY) followed by G3b (28.7 PKPY), G3a (21.1 PKPY), and G2 (15.9 PKPY). There were low numbers of events in the T1DM cohort and the G5 categories, therefore, the crude rates should be interpreted with care. When stratified by proteinuria, people with a proteinuria category of A2/3 (increased) and A3 (severely increased) had similar rates of admissions in non-DM (10.4 vs. 9.1 PKPY, respectively) and in T2DM (20.2 vs. 18.5 PKPY, respectively).

**Table 2:** Hospital admission rates for MACE by eGFR category at baseline and by proteinuria category nearest to admission date, by diabetes cohort.

	Non-DM		T1DM		T2DM		Overall	
	n	Rate PKPY	n	Rate PKPY	n	Rate PKPY	n	Rate PKPY
**eGFR category at baseline**								
G1								
G2	3,480	11.2	7	9	841	15.9	2,607	28.4
G3a	2,888	14.4	0	0	370	21.1	3,881	41.8
G3b	1,125	21.0	0	0	142	28.7	2,201	51.9
G4	219	27.3	0	0	37	43.8	506	82.6
G5	29	32.4	0	0	0	0	28	49.2
Overall	7,763	13.5	8	6.1	1,485	18.4	9,256	39.3
**Proteinuria category nearest MACE admission date**								
A1	939	4.3	1	1.8	485	12.4	1,425	18.6
A2	396	6.0	1	4.1	302	14.3	699	24.3
A2/3	40	10.4	0	0.0	49	20.2	89	30.6
A3	132	9.1	2	6.4	179	18.5	313	34.0
Missing	6,256	22.9	4	24.5	470	55.0	6,730	102.5
Overall	7,763	13.5	8	6.1	1,485	18.4	9,256	39.3

Non-DM, non-diabetes; T1DM, type 1 diabetes; T2DM, type 2 diabetes; PKPY, per thousand patient years; KDIGO eGFR categories: G1, normal or high (≥90 ml/min/1.73m2); G2, mildly decreased (≥60 and <90 ml/min/1.73m2); G3a, mildly to moderately decreased (≥45 and <60 ml/min/1.73m2); G3b, moderately to severely decreased (≥30 and <45 ml/min/1.73m2); G4, severely decreased (≥15 and <30 ml/min/1.73m2); G5, kidney failure (<15 ml/min/1.73m2); proteinuria categories: proteinuria categories: A1, normal to mildly increased; A2, moderately increased; A3, severely increased; A2/3, increased.

People with a proteinuria category of A2 (moderately increased) had a crude rate of 6.0 PKPY in non-DM and 14.3 PKPY in T2DM. People with a proteinuria category of A1 (normal to mildly increased) had a crude rate of 4.3 PKPY in non-DM and 12.4 PKPY in T2DM (Table 2).

## Discussion

This study characterized the influence of eGFR and proteinuria, two commonly used metrics of renal disease morbidity, on the risk of progression to major adverse cardiovascular event (MACE) and found both to be directly and independently associated with increased risk. This was evident both in non-diabetes and in type 2 diabetes, with insufficient follow-up in people with type 1 diabetes. However, the adjusted risk of progression to MACE in the same eGFR category was between 4.6 and 2.4 times higher in those with type 2 diabetes than in those with no diabetes, depending upon where the comparison was made, with this ratio decreasing overall as renal function declined.

The patterns of risk change by eGFR category followed a similar pattern in both the type 2 diabetes and non-diabetes cohorts, with aHRs increasing as eGFR function decreased from G2 to G5. It appeared, in crude analysis, that the type 1 cohort had a higher risk of MACE at eGFR category G5 (kidney failure) than did the type 2 diabetes cohort, but the type 2 diabetes cohort had a higher overall risk of MACE than did the type 1 diabetes cohort, which may reflect the former’s higher mean age, BMI, and duration of diabetes. This increased risk of MACE as eGFR function decreases is similar to findings by Kottgen and colleagues,[27] where the adjusted risk of heart failure was 76% higher in persons with moderately or severely decreased kidney function (categories G3a–G5) compared with those with mildly decreased function (category G2). A *post-hoc* analysis by gender showed that the pattern of increased risk of MACE by eGFR severity differed between females and males; however, further research is needed to understand the factors potentially increasing risk in women with kidney failure.

Increased severity of proteinuria was also independently associated in this study with increased adjusted risk of progression to MACE in both the type 2 diabetes and non-diabetes cohorts. A similar pattern was evident when models were applied adjusting for the interaction between eGFR and proteinuria in the overall, merged cohort: the highest risks of progression to MACE were associated with the more severe combinations of eGFR and proteinuria category. This finding resembles those of a meta-analysis by Fox and colleagues,[28] in which increasingly severe combinations of decreased kidney function and higher proteinuria were associated with an increased risk of cardiovascular mortality in both diabetic and non-diabetic individuals. (In our study, surprisingly, the most severe combination of all, G5/A3, was associated with only the second-highest risk of MACE, after the combination G5/A1; however, this may have been an artefact of low numbers and consequent wide confidence intervals for the latter combination.)

Morbidity in the kidney leads to cardiovascular morbidity, and vice versa. Thus, morbidity in these two vital organs is closely interrelated. Disease risk in both organs is also affected by hyperglycaemia from diabetes, although more so in the kidney. These interrelationships are therefore complex and involve an interplay between metabolic diseases, microvascular disease, and macrovascular disease. The complex morbid interaction is due, in all likelihood, to a clustering of risk factors, including ageing, high blood pressure, and dyslipidaemia.[29] Other risk factors may also play a part, such as anaemia, volume overload, and systemic inflammation. Haemodynamic abnormalities can activate renal inflammation leading to fibrosis, which may then in turn result in cardiovascular morbidity. This is exemplified by the statistic that in people with end-stage renal disease treated with dialysis, the risk of cardiovascular mortality is increased 10- to 20-fold compared with those without CKD.[30] Furthermore, angiotensin-converting enzyme (ACE) inhibitors and angiotensin II receptor blockers (ARBs) commonly used to for renoprotection and in the management of hypertension and heart failure have complex effects on renal function via the renin–angiotensin system. All these factors, and others, have an impact on the patterns of association that we report. Nevertheless, these general patterns of association appear to be both clear and predictable and demonstrate an unmet need to reduce cardiovascular risk in all people with renal disease regardless of their diabetes status.

Where people have type 2 diabetes, some of the newer antidiabetes drugs, the SGLT2 inhibitors and GLP-1 receptor agonists, have demonstrated cardiovascular benefit and well as some benefits on renal outcomes.[31] Taken together, the results from this study suggest that may be a place for these and other therapies in the management of diabetics with renal disease given their excess risk of cardiovascular outcomes.

There are strengths and limitations that can be drawn from the study design and data source. Routine observational data have the merit of including large numbers of people in a real-world setting. However, both eGFR and proteinuria measurements could be taken at any time, and observations are therefore sometimes understandably sparse and lacking in structure, which may introduce variability. Proteinuria was not measured in a standard way, and therefore we had to distil the various methods of recording into broad categories, creating a new category (A2/3) where the extent of proteinuria increase was not known. Patients with no proteinuria measurements in our data progressed more rapidly to MACE than did patients with known proteinuria status, and this may be because patients in the former group had more advanced renal morbidity and were being predominantly monitored in secondary care, the test data from which are not included in our data source. We may have identified people with diabetes whose kidney disease had a non-diabetic aetiology. We did not adjust for antihypertensive or antidiabetic medication in our analyses. There were low numbers of people in the type 1 diabetes cohort and also in the G5 category, and findings from the G5 category in particular need to be interpreted with caution. Although used commonly, MACE is a composite clinical endpoint that includes both cardiovascular and cerebrovascular disease events.

## Conclusions

This population-based study demonstrated that the risk of MACE increased with increasing disease severity within non-diabetes and type 2 diabetes cohorts in relation to renal morbidity as measured by eGFR and proteinuria. These two metrics were independent risk markers.

The observation that reduced eGFR and increased proteinuria appear synergistic with respect to risk of ischaemic cardiovascular events is of significant concern. Treatment intensification to achieve control of hypertension by means of agents such as ACE inhibitors and spironolactone that act via the renin–angiotensin system is clearly warranted both to preserve renal function and reduce protein loss.

We have also demonstrated that individuals with type 2 diabetes have a much higher risk of MACE than those without diabetes after accounting for eGFR level. Intensified glycaemic control with agents that are known to reduce the risk of cardiovascular events but not significantly increase the risk of hypoglycaemia, (such as SGLT-2 inhibitors and GLP-1 receptor agonists) may also be important with respect to preserving renal function.

## Supporting information

S1 Table eGFR categories. (DOCX)

S2 Table Proteinuria categories. (DOCX)
