# Supplementary material for: Major adverse cardiovascular events in people with chronic kidney disease in relation to disease severity and diabetes status
Source: PLoS One. 2019 Aug 28;14(8):e0221044. doi: 10.1371/journal.pone.0221044 (PMC6713399; doi:10.1371/journal.pone.0221044)
Supplement: S1 Table — (DOCX) [file pone.0221044.s001.docx]

**Major adverse cardiovascular events in people with chronic kidney disease in relation to disease severity and diabetes status**

Craig J. Currie, Ellen Berni, Thomas R Berni, Sara Jenkins-Jones, Marvin Sinsakul, Lutz Jermutus, Philip Ambery, Meena Jain

**Supporting Information**

**S1 Table.** **eGFR categories**

| eGFR category | Description | Range (ml/min/1.73m^2^) |
| --- | --- | --- |
| G1 | Normal or high | ≥ 90 |
| G2 | Mildly decreased | ≥ 60 and < 90 |
| G3a | Mildly to moderately decreased | ≥ 45 and < 60 |
| G3b | Moderately to severely decreased | ≥ 30 and < 45 |
| G4 | Severely decreased | ≥ 15 and < 30 |
| G5 | Kidney failure | < 15 |
