# Supplementary material for: Major adverse cardiovascular events in people with chronic kidney disease in relation to disease severity and diabetes status
Source: PLoS One. 2019 Aug 28;14(8):e0221044. doi: 10.1371/journal.pone.0221044 (PMC6713399; doi:10.1371/journal.pone.0221044)
Supplement: S2 Table — (DOCX) [file pone.0221044.s002.docx]

**Major adverse cardiovascular events in people with chronic kidney disease in relation to disease severity and diabetes status**

Craig J. Currie, Ellen Berni, Thomas R Berni, Sara Jenkins-Jones, Marvin Sinsakul, Lutz Jermutus, Philip Ambery, Meena Jain

**Supporting Information**

**S2 Table.** **Proteinuria categories**

| Category | Description | Qualitative signifiers | Albumin–creatinine ratio, mg/mmol | Albumin excretion rate, mg/24hours | Spot albumin,  mg/l | Protein–creatinine ratio, mg/mmol | Protein excretion rate, mg/24hours | Spot protein,  mg/l |
| --- | --- | --- | --- | --- | --- | --- | --- | --- |
| A1 | Normal to mildly increased | Normal, no abnormality, negative, trace | < 3 | < 30 | < 30 | < 15 | < 150 | < 150 |
| A2 | Moderately increased | Microalbuminuria | 3 to 30 | 30 to 300 | 30 to 300 | 15 to 50 | 150 to 500 | 150 to 500 |
| A3 | Severely increased | Macroalbuminuria, proteinuria | > 30 | > 300 | > 300 | > 50 | > 500 | > 500 |
| A23 | Increased | Abnormal, positive, high, albuminuria | ≥ 3 | ≥ 30 | ≥ 30 | ≥ 15 | ≥ 150 | ≥ 150 |
